# Laparoscopic Unroofing and Aspiration-Sclerotherapy in the Management of Symptomatic Simple Renal Cysts

**DOI:** 10.1100/tsw.2006.358

**Published:** 2006-01-19

**Authors:** Serdar Arisan, Ayhan Dalkilinc, Turhan Caskurlu, Nurettin Cem Sonmez, Soner Guney, Erbil Ergenekon

**Affiliations:** Sisli Etfal Research and Training Hospital, 1st Urology Clinics, Sisli-Istanbul, Turkey

**Keywords:** sclerotherapy, unroofing, renal cysts

## Abstract

Simple renal cysts are quite common in adults with an incidence that increases with age. Sclerosant treatment is very common, but the recurrence rate is high. Results are still under investigation for laparoscopic approaches and their long follow-up periods. Between 1998 and 2004, 21 patients were diagnosed with symptomatic renal cysts in our clinics. Initially, all patients underwent aspiration-sclerotherapy with 95% ethanol, the most common sclerosant, under ultrasound, fluoroscopy, or CT guidance. For those with sclerosant therapy failure, the laparoscopic unroofing method was used. Like open surgery, laparoscopic unroofing of the cyst appears to be effective by not only removing part of the cyst wall, but more importantly, by providing adequate drainage of the cyst. After sclerotherapy, 71% of the patients had recurrent pain and cyst on follow-up (at mean 14 months). This group of patients was cured with the laparoscopic unroofing method and there is still no recurrence.We emphasize the unroofing method as better than single session sclerotherapy. And also, laparoscopic unroofing of the cyst is more predictable and has better results than sclerotherapy aspiration.

## BACKGROUND

Simple renal cysts are quite common in adults with an incidence that increases with age[1,2]. They are generally diagnosed incidentally on sonography, computed tomography (CT) scans, or urography performed for urinary tract or other abdominal problems[3].

Asymptomatic renal cysts are best left alone. However, renal cysts may gestate a number of symptoms. These include flank pain and hematuria, hypertension, repeated infections, and pelvicalyceal system obstruction (rarely). The treatment options for symptomatic cysts include aspiration, aspiration and instillation of sclerosing agents, percutaneus marsupialization, and open or laparoscopic unroofing[4,5]. During the last decade, many laparoscopic advances have been developed in the field of urology. The laparoscopic approach has also been used to treat symptomatic renal cysts definitively as a minimally invasive alternative[6,7].

A symptomatic large renal cyst may be dealt with in a variety of ways, the simplest of which is needle aspiration. A significant proportion of cysts treated in this fashion will recur[8]. The excellent results achieved with laparoscopic decortications of symptomatic simple renal cysts were first described by Hulbert[9]. For over 5 years, we have been interested in minimally invasive management of symptomatic simple renal cysts. We initially managed these patients with ethanol sclerotherapy, but because the results were poor, we changed the laparoscopic cyst ablation. In this report, we evaluate the efficacy and morbidity aspiration-sclerotherapy vs. laparoscopic surgical techniques in one institution for a 10-year period[10].

## MATERIAL AND METHODS

The difference in outcomes was retrospectively examined in the case histories of patients who presented with chronic flank or lumbar pain refractory to medical management for treatment for symptomatic renal cysts between 1998 and 2004 in our clinics; 21 patients (mean age 60 years; range 42–77; 11 men and 10 women) with symptomatic, voluminous renal cysts were entered to this study. Continuous lumbar pain (18 patients), renal colic without evidence of other urologic disease (3 patients), hypertension (4 patients), and simple cyst causing pelvicalyceal obstruction (15 patients) were accepted as treatment indications. All patients were studied with ultrasound, excretory urography, and CT scans. In this series, all cysts were of type I according to the Bosniak classification.

Initially, all patients underwent aspiration-sclerotherapy with 95% ethanol, the most common sclerosant, under ultrasound, fluoroscopy, or CT guidance. After local anesthetic infiltration, a Kellett catheter needle was used for puncturing the cyst. The technique was based on ultrasound-guided percutaneous puncture of the cyst with an 18-gauge needle and positioning of a pigtail side-hole 5-Fr catheter, according to the Seldinger technique. A pigtail catheter was then inserted over a guide wire and the cyst was aspirated to dryness. Each cyst was treated with different catheters in patients who presented with single and multiple cysts.

A sample of the fluid obtained during the initial aspiration was sent for cytological examination in all cases. Only simple cysts on ultrasonography or CT, with no communication with the pelvicalyceal collecting system on a contrast medium study, were considered for sclerotherapy. Contrast medium was instilled into the cyst to ensure that there was no communication with the pelvicalyceal collecting system, and to exclude any leakage from the puncture site. After evacuation of contrast medium, 95% ethanol was injected (approximately 20% of cyst volume and up to maximum 75 ml). This was left indwelling for 20 min; the patient was asked to change position every 5 min to make the sclerosant touch the cyst walls. The ethanol was then reaspirated completely. After sclerotherapy, patients were observed overnight. Ultrasonography was repeated 6–12 weeks after treatment. The patients were followed up within 3 months of the procedure and 6–12 months afterwards. In the follow-up process, the recurrence was determined for those patients who suffered with the same problems (nearly the same-dimension voluminous cyst causing obstruction, hypertension, continuous lumbar pain, or renal colic without evidence for other urological diseases) after treatment. In recurrent patients, a retroperitoneal laparoscopic unroofing technique was used (Fig. 1). All patients initially were given a simple diagnostic aspiration test to check the communication between a cyst and the pelvicalyceal collecting system, potentially leading to a retroperitoneal urinoma or urinary ascites if laparoscopic unroofing was used alone. The patient was successfully managed by retroperitoneoscopic unroofing using a three-port technique. Under general anesthesia, the patient was catheterized and positioned in the left/right semi-lateral position. The operator stood at the side of the patient's back. The first port site was inserted 2 cm below and lateral to the tip of the 12th rib. A 1.0-cm skin incision was done on the lumbar triangle (Petit’s). A Visiport optic trocar was introduced under camera vision. The optic cutting device was deepened layer by layer, passing through the subcutaneous layers, muscle layers, and lumbodorsal fascia and finally entering the retroperitoneal space. The Visiport cutting device was removed. A 0-degree laparoscope was inserted through the sheath.

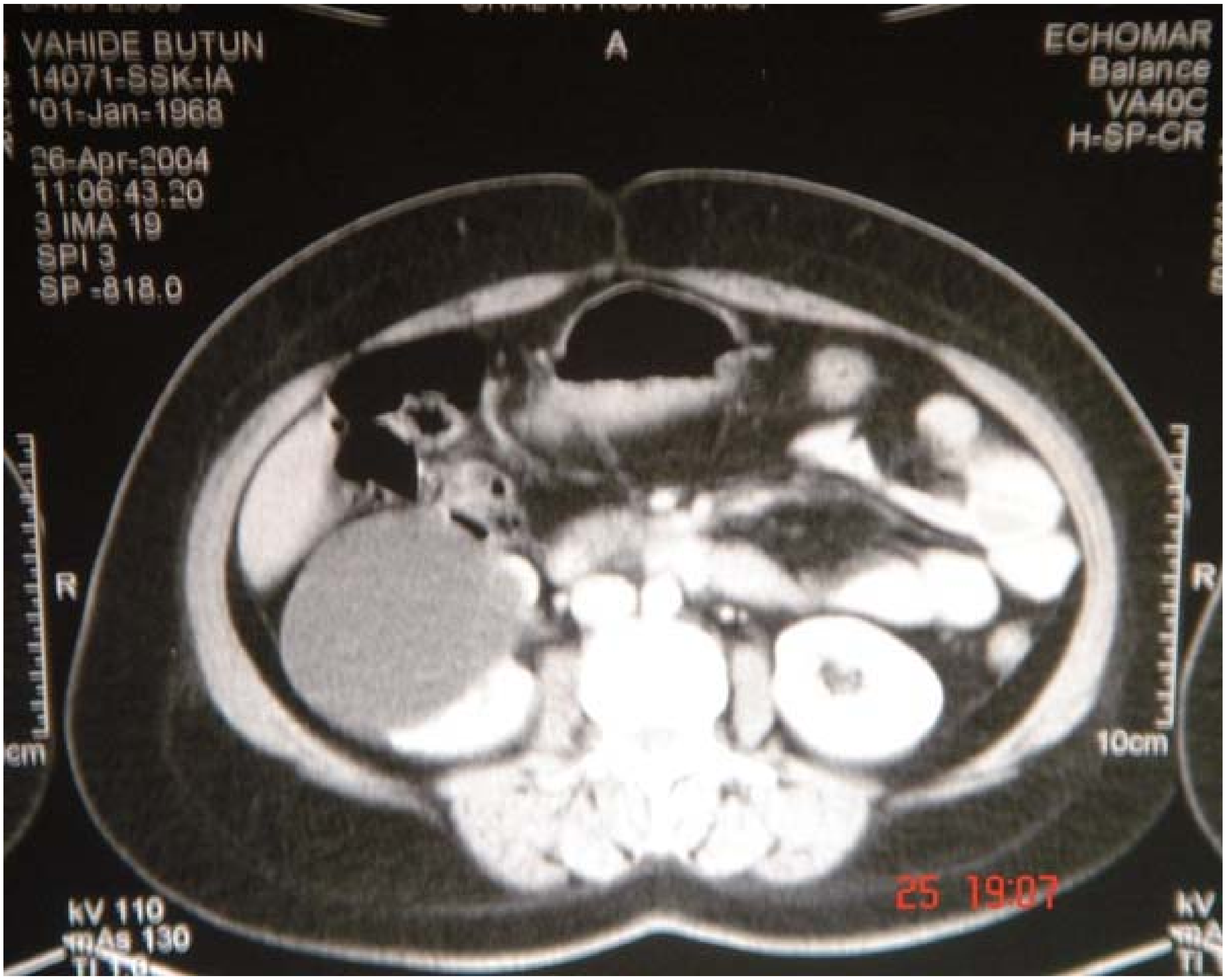

**FIGURE 1.** CT demonstrated the preoperative renal cyst after monosession ethanol sclerotherapy.

Carbon dioxide was insufflated to 15 mmHg and the laparoscope with pneumodissection was advanced into the retroperitoneal space. The direction of dissection depended on the location of the renal cyst (anterior, posterior, or central aspect; upper, middle, or lower pole). The laparoscope was removed. Then, a modified balloon dissector was made with the middle finger of a No. 7.5 latex surgeon glove. It was cut out and tightened to the tip of an 18 French Robison tube. The balloon dissector was put into the retroperitoneum via the trocar sheath. It was inflated with normal saline by Toomey syringe to 500–700 ml to create working space. The balloon dissector was deflated and removed. The laparoscope was again inserted. The second 5-mm trocar was inserted under vision through a posterior axillary line roughly posterolateral to the kidney, allowing a grasping forcep to be inserted. The third 10-mm trocar was inserted in the anterior axillary line approximately anterolateral to the kidney; via that trocar, a dissecting forcep or clip applier was inserted. The Gerota's fascia was opened and the kidney was identified. The cyst was dissected and the edge of the cyst was delivered. The cystic fluid was obtained and then a suction-irrigation cannula was inserted via the posterior port to remove fluid. The cystic wall was excised with and the edge sealed by electrocautery. The base of the cyst was inspected to exclude any tumor growth. The perirenal fat was placed over the base of the cyst. The 18 French Robison tube previously used for the balloon dissector was positioned via a laparoscopic port for drainage. All the ports were removed. The wounds were closed in layers, with 2-0 vicryl for the muscle-fascia, with 4-0 vicryl for the subcuticle, and 3M steri-stripe for the skin. The follow-up period for these laparoscopically treated patients is still ongoing.

## RESULTS

Of the patients who were cured with only sclerotherapy, 5 of 21 had only minor complications such as abdominal discomfort, low-grade fever, or local pain; the symptoms disappeared spontaneously or after treatment with analgesics. One patient had blood in the drained fluid that disappeared by the end of treatment.

Preoperative CT revealed a single cyst in 16 patients, two cysts in 4 patients, and four cysts in 1 patient. None of the patients were noted to have peripelvic cysts. Patients' characteristics and outcomes are summarized in Table 1. All cyst fluid cytological analysis was negative and not malignant as expected before. Of the patients, 15 of 21 (71%) had recurrent pain and cyst on follow-up (at mean 14 months; range 3–20 months). Therefore, the laparoscopic unroofing technique applied to all these patients. The mean (range) operation duration (excluding anesthetic working time) was 96 (85–104) min. The mean operative blood loss was 127 ml. The hospital stay afterward was 3 (2–7) days. There was no specific complication in laparoscopic-managed cases. All were cyst free at the last follow-up, at a mean (median, range) of 25 (10–48) months.

**TABLE 1**
**Comparison of Aspiration-Sclerotherapy and Laparoscopic Unroofing Applied Patient Characteristics**

| Description | Aspiration-Sclerotherapy | Laparoscopic Unroofing |
|---|---|---|
| Number of patients | 21 | 15 |
| Mean age (range), years | 60 (42–77) | 57 (45–68) |
| Sex | 11M, 10F | 6M, 9F |
| Presentation | | |
| Loins pain | 21 | 15 |
| Flank mass | None | None |
| Median range | | |
| Cyst diameter (cm) | 6 (5.2 – >16.4) | 9 (5–15) |
| Cyst vol. aspirated (ml) | 86 (25–136) | 250 |
| Mean (range) follow-up, months | 14 (3–20) | 25 (10–48) |

## DISCUSSION

Renal cysts are mostly asymptomatic and only rarely can regress spontaneously. Large or multiple cysts may become clinically evident because of the compression of the renal pelvis or adjacent organs. Symptoms include abdominal discomfort or pain, hypertension secondary to vascular compression, and, rarely, renal failure[7]. Only symptomatic cysts, larger than 5 cm, are usually treated. Percutaneous management of renal cysts is the easiest and fastest procedure, and no major complications are generally encountered. Several techniques have been proposed for the treatment of symptomatic simple renal cyst. Aspiration alone has been used in the treatment of polycystic renal disease, but it is associated with a high rate of recurrences (78–100%); other techniques include aspiration and injection of a sclerosant with or without biologic glue (recurrence rate 30–40%) and percutaneous resection of the cyst wall[11,12,13]. Percutaneous sclerotherapy is usually used as the first-line treatment before surgical or laparoscopic decompression of simple renal cysts that require treatment[14]. It is clearly better than aspiration alone, where the cyst fluid almost always reaccumulates. Of the various sclerosants, 95% ethanol was used in the present patients because it rapidly (1–3 min) coagulates the cells lining the cyst, but slowly penetrates the fibrous capsule (4–12 h), causing minimal local or systemic complications[7]. A small-caliber pigtail catheter was used in the present patients, with no tract dilatation, and there was no damage to the collecting system or adjacent organs. Mild and transient fever and flank discomfort were the only minor complications.

With the advent of modern imaging methods, most cysts are aspirated as part of a therapeutic maneuver in symptomatic patients[6,8]. This method has the advantages of being minimally invasive, is performed under local anesthetic, has a short period of hospitalization, and is cheap compared with open surgical treatment of the cyst. Although several authors reported on the safety and high success rate of multiple-session aspiration and sclerotherapy[12,15], we did not apply this treatment to our recurrent cases after single session sclerotherapy.

Published reports suggest that cyst recurrence after sclerotherapy is a result of inadequate sclerosis of the cyst wall epithelial cells, which then continue to produce fluid into the cyst[7]. The needle puncture site for sclerotherapy appears to close soon afterwards, and so does not provide drainage of the cyst. In our study, after sclerotherapy, pain was observed in some patients. It was associated with recurrence of the cysts. Although occurring after ethanol instillation, it was not because of sclerosant usage.

In 1998, we began ethanol sclerotherapy because it was the most commonly reported method for renal cysts, with a good safety record[16]. However, its results had failure for many patients (see Table 1). Therefore, we changed the treatment model for recurrent patients. Although high success rates have been reported for multiple-session ethanol instillation treatment, with some concerns about safety in the methods described, we preferred laparoscopic treatment as an attractive alternative. Laparoscopic techniques have the advantages of a minimally invasive procedure with the effectiveness of cyst marsupialization achieved by open surgery. Several authors reported on the technique and success of this method[17,18,19]. We adopted this approach after disappointing results with sclerotherapy and, as shown in Table 1, the results are very encouraging so far. Like open surgery, laparoscopic unroofing of the cyst appears to be effective by not only removing part of the cyst wall, but more importantly, by providing adequate drainage of the cyst. We consider that our method of selecting patients contributed to the success. The follow-up period results showed that the unroofing method is better than monosession sclerotherapy. The follow-up period is still ongoing and there is no recurrence after the unroofing operation. However, a longer follow-up period and a large series of patients will be useful for validation of this approach.

## CONCLUSION

A rigorous clinical evaluation is advised before ascribing any loin pain to a simple renal cyst found on imaging; the response to simple aspiration is part of that evaluation. When symptomatic cysts are established and a definitive treatment of a cyst is indicated, laparoscopic unroofing of the cyst is more predictable and has better results than sclerotherapy aspiration.

## ACKNOWLEDGMENTS

We thank Damla Buyuktuncer (PhD) for editorial help.
